# Cement Equivalence of Metakaolin for Workability, Cohesiveness, Strength and Sorptivity of Concrete

**DOI:** 10.3390/ma13071646

**Published:** 2020-04-02

**Authors:** J.J. Chen, Q.H. Li, P.L. Ng, L.G. Li, A.K.H. Kwan

**Affiliations:** 1 Department of Civil Engineering, Foshan University, Foshan 528000, China; 2 Faculty of Civil Engineering, Vilnius Gediminas Technical University, LT-10223 Vilnius, Lithuania; 3 Department of Civil Engineering, The University of Hong Kong, Hong Kong 999077, China; khkwan@hku.hk; 4 School of Civil and Transportation Engineering, Guangdong University of Technology, Guangzhou 510000, China; ligu@gdut.edu.cn

**Keywords:** cement equivalent factor, cohesiveness, metakaolin, sorptivity, strength, workability

## Abstract

A series of concrete mixes with metakaolin (MK) content ranging from 0 to 30% and water/cementitious materials (W/CM) ratio varying from 0.30 to 0.50 were produced for performance testing. The results showed that adding MK up to 20% as ordinary Portland cement (OPC) replacement best improved the 28-day and 70-day cube strengths, whereas adding MK up to 30% as OPC replacement always increased the cohesiveness and decreased the sorptivity, but impaired the workability. Moreover, the cement equivalent factor (CEF), i.e. the equivalent mass of OPC per mass of MK added, for each performance attribute, including workability and cohesiveness, was evaluated. Whilst the actual CEF of MK was generally higher at a higher W/CM ratio and lower at a higher MK content, overall, the average CEFs were found to be 1.98, 2.17, 3.83, 1.93, 2.12, and 4.70 for slump, flow, cohesiveness, 28-day cube strength, 70-day cube strength, and sorptivity coefficient, respectively. These CEF values indicated that the MK is a highly effective cementitious material for improving the cohesiveness, strength, and durability. Moreover, it has been demonstrated that the CEFs for workability and cohesiveness are useful parameters in aiding the mix design of MK concrete.

## 1. Introduction

Supplementary cementitious material (SCM) is commonly utilized in the production of high-performance concrete (HPC). Aїtcin [1] suggested that the particle size of SCM for HPC should be 0.5 to 5.0 μm, which is finer than that of cement, for the sake of improving the packing density and avoiding too much of an increase in the surface area of the binder phase. Among the various SCMs, metakaolin (MK), which is an anhydrous calcined form of kaolinite generally ground to finer than cement, should be a good choice [2,3,4]. As a thermally activated amorphous mineral, MK is acquired through calcining kaolin clay at 700–800°C, during which the kaolinite is dehydroxylated [5,6]. With appropriate control of the process and quality, MK can be produced to have high uniformity and high purity [7]. Chemically, MK reacts with the calcium hydroxide that was generated from cement hydration to form secondary calcium silicate hydrate [8,9,10]. Physically, MK functions as a micro-filler that fills into the interstitial space between cement particles to densify the solid skeleton of the binder phase [11].

The utilization of MK in concrete has attracted great research interest due to the high effectiveness of MK as a SCM. For the effect of MK on workability, Hassan et al. [12] found that the addition of MK increased the viscosity and improved the passing ability of the concrete mix. Nazário Santos et al. [13] reported that the addition of MK increased the yield stress and the thixotropic impedance of the cement paste. This was because the MK particles, by filling into the gaps between the larger size cement particles, introduced additional inter-particle forces inside the paste. Chu and Kwan [11] demonstrated that the addition of MK, together with a high superplasticiser dosage, increased the workability, but it sometimes increased or decreased the degree of leveling. Muduli and Mukharjee [14] reported that the addition of MK slightly reduced the workability of recycled coarse aggregate concrete. Nguyen Amanjean et al. [15] showed that the addition of MK offered fiber reinforced concrete higher plastic viscosity, thixotropy and structuration rate (i.e. less time being required to gain the structure during rheological flow).

For the effect of MK on strength, Justice and Kurtis [16] measured that the addition of MK increased the percentage of sub-10 nm pores to the total pores, and advocated that the refinement in microstructure caused the improvements in strength and impermeability. Cassagnabère et al. [17] reported that the addition of MK up to 25% improved the one-day strength and maintained the 28-day strength of steam-cured precast concrete. Güneyisi et al. [18] showed that the addition of MK enhanced both the strength development rate and the long-term strength at various water/cementitious materials (W/CM) ratios. Hassan et al. [12] showed that the addition of MK always increased the compressive strength of concrete. Valipour et al. [19,20] showed that the addition of MK always increased both the compressive strength and electrical resistivity of concrete. Muduli and Mukharjee [14] demonstrated that the addition of MK was able to compensate for the reductions in compressive strength, flexural strength, and splitting tensile strength due to the use of recycled coarse aggregate. All of these results indicate that MK is better than most other SCMs, such as fly ash and slag.

For the effect of MK on durability, Güneyisi et al. [18] discovered that the addition of MK lowered the apparent gas permeability of concrete and decreased the shrinkage crack width. Hassan et al. [12] revealed that the addition of MK reduced the shrinkage and chloride permeability, and enhanced the resistances to freezing and thawing as well as scaling. Moreover, the beneficial effect of MK on the voids pattern was more pronounced than that of micro-silica. Valipour et al. [19,20] showed that the MK decreased the chloride diffusion, but it had little effect on the water absorption. On magnesium sulphate attack, Kiachehr and Omid [21] found that brucite would be formed to cause cracking. Kavitha et al. [22] attributed the higher sulphate resistance to the reduced ettringite, brucite, and gypsum formation, the lower permeability and chloride ingress to the refined pore structure, and the better acid resistance to the larger consumption of calcium hydroxide through pozzolanic reaction. Tafraoui et al. [23] showed that concrete with MK could have excellent resistance to carbonation and chloride attack. Muduli and Mukharjee [14] reported that the addition of MK reduced the voids volume and the water absorption. Saboo et al. [24] proved that the addition of just 2% MK could result in 10% lower porosity of the concrete.

For the roles of MK in concrete construction, Tafraoui et al. [25] pointed out that the wide availability and affordable price of MK enhanced its promising potential in producing ultra-high performance concrete. Additionally, the white colour of MK could render an aesthetic advantage in architectural surface finishing. Kavitha et al. [22] remarked that the use of MK in concrete would consume less thermal energy and generate less CO_2_. Chen et al. [26] showed that the addition of MK could reduce the energy consumption through lowering the curing temperature in the manufacturing of steam-cured concrete. Furthermore, by adding MK to make the structure more durable, the resulting structure will require less maintenance and repair throughout the life-cycle, and, thus, will have longer service life for higher sustainability.

On the other hand, for quantitative evaluation of the cementing efficiency of a SCM, the concept of cementing efficiency factor (CEF), which is defined as the mass of cement that can be substituted per unit mass of SCM added without affecting the strength or durability attribute under investigation, has been advocated for years [27,28,29]. This concept has been applied to evaluate the cementing efficiencies of various SCMs solely with respect to the strength and durability performances [11,30,31,32,33,34,35,36,37,38]. Herein, as the effects of the SCM on the workability and cohesiveness will also be investigated, the term “cementing efficiency factor” is changed to “cement equivalent factor” because the workability and cohesiveness are un-related to the cementing efficiencies. It is defined as the equivalent mass of cement per unit mass of SCM added without affecting the performance attribute under investigation and is also abbreviated as CEF.

Up to now, most previous studies on the CEFs of MK were rather limited and the concept of CEF has not been applied to fresh properties. Additionally, as different researchers used different types of MK, widely different results have been obtained [18,23,39,40,41,42,43,44,45]. In this study, the CEFs of MK for different performance attributes, including workability, cohesiveness, strength, and durability were evaluated via a systematic research programme where trial concrete mixes with varying MK content and W/CM ratio were produced for performance evaluation. Apart from evaluating the CEFs of MK, the concurrently achieved performances were studied to develop a mix design strategy for the use of MK in concrete.

## 2. Materials and Research Design

### 2.1. Materials

Two types of cementitious materials, namely: OPC (ordinary Portland cement) and MK (metakaolin) were utilized. The OPC was of type CEM I 42.5N and complied with EN 197-1: 2011 [46]. The MK was obtained from a clay calcination factory in China and complied with Chinese National Standard GB/T 18736: 2017 [47] and Yunnan Provincial Standard DB 53/T 843: 2017 [48]. The fine and coarse aggregates were both crushed granitic rock. Their nominal maximum sizes were 5 and 20 mm, respectively. Sieve analysis verified that the grading curves of the fine and coarse aggregates were within the allowable limits that were stipulated in BS 882: 1992 [49]. The OPC, MK, and aggregates were, respectively, produced in Jiangxi Province, Inner Mongolia Province and Guangdong Province in China. The relative densities of the OPC, MK, fine and coarse aggregates were measured as per BS 812-2: 1995 [50] as 3.10, 2.59, 2.48, and 2.61, respectively.

**Figure 1:**
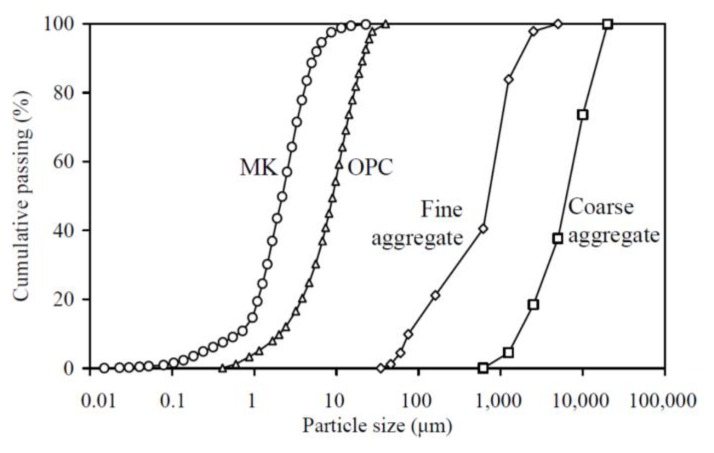
Particle size distributions of the raw materials.

**Figure A1:**
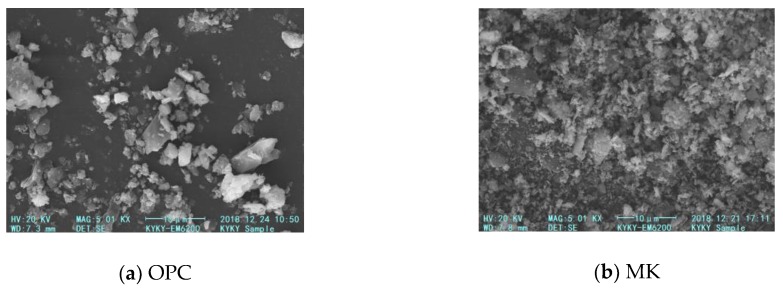
Scanning electron micrographs of OPC and MK.

The particle size distributions of the OPC and MK were measured with a laser diffraction particle size analyzer, and the particle size distributions of the fine aggregate and coarse aggregate were measured by mechanical sieving, as depicted in Figure 1. The volumetric mean particle sizes of the OPC, MK, fine aggregate, and coarse aggregate were 9.34 μm, 3.20 μm, 0.78 mm, and 7.24 mm, respectively, as calculated from the particle size distributions. The chemical compositions of the OPC and MK were obtained through the X-ray diffraction method, as displayed in Table 1, and their micrographs were obtained through scanning electronic microscopy, as displayed in Figure A1 in the Appendix. The particle size distributions and micrographs both suggested that the MK is finer than OPC, such that the MK particles can fill into the interstitial space between the OPC particles to achieve denser packing of the solid skeleton.

As in the production of high-strength and high-performance concretes, a polycarboxylate-based superplasticiser was added to all of the concrete mixes in the experimental programme. The adopted superplasticiser was imported from Germany. It was an aqueous solution with a relative density of 1.03. It functioned by dispersing the fine particles and reducing their agglomeration, so that the cementitious materials, including the MK, are more evenly dispersed in the binder phase [51]. 

### 2.2. Concrete Mixes

A series of 20 concrete mixes were produced for testing. To reveal the effects of MK on the various performance attributes, 16 concrete mixes with the MK content varying from 0% to 30% by volume with a step of 10% and the W/CM ratio varying from 0.30 to 0.50 by mass with a step of 0.10 were incorporated. Four additional concrete mixes without MK at W/C ratios of 0.25, 0.35, 0.45, and 0.55 by mass were incorporated to obtain the relationship between each performance attribute and the water/cement (W/C) ratio. Hence, there were a total of eight concrete mixes without MK for correlating each performance attribute to the W/C ratio. For all of the concrete mixes, the cementitious paste volume (i.e. the volume of water and cementitious materials as a percentage of volume of concrete) was fixed at 38%. As a result, the aggregate volume (i.e. the volume of aggregate as a percentage of volume of concrete) was fixed at 62%. The fine aggregate to total aggregate ratio was fixed at 0.40 by mass. Regarding the superplasticiser dosage, it was set at 3.0% by mass of the cementitious materials, the maximum dosage for OPC concrete in normal circumstances, as recommended by the supplier.

In terms of quantity per volume of concrete, the fine aggregate content and coarse aggregate content were fixed at 634.0 and 951.0 kg/m^3^, respectively. The OPC content varied from 297.9 to 663.7 kg/m^3^, the MK content varied from 0 to 156.7 kg/m^3^, the water content (including the water in the superplasticiser) varied from 165.9 to 247.1 kg/m^3^, and the superplasticiser content varied from 12.1 to 19.9 kg/m^3^. Table 2 lists the detailed mix proportions. Each concrete mix was assigned an identification code in the format of M-V_MK_-W/CM, in which M denotes MK concrete and V_MK_ denotes the MK content.

## 3. Testing Methods

Since the tests in this study are standardized or commonly adopted tests for workability, cohesiveness, strength, and durability measurement, only brief outlines are given herein. Details of the testing procedures have been reported elsewhere [52,53,54].

### 3.1. Slump and Flow Measurement

The slump-flow test was conducted following EN 12350-2: 2009 [55]. Before the test, a level, smooth, and flat steel plate large enough for the concrete patty formed was placed on the floor. The concrete sample was filled into the slump cone with the surplus removed and top surface trowelled flat. The slump cone was steadily lifted, such that the concrete deformed and flowed by gravity. The drop in height and the average diameter of the patty formed were taken as slump and flow, which were respectively taken as measures of deformability and flowability. Any significant segregation, as indicated by the presence of a strip of mortar without coarse aggregate at the edge of the concrete patty, was recorded.

### 3.2. Segregation Index Measurement

The sieve segregation test was conducted following EN 12350-11: 2010 [56]. The concrete sample was poured onto a 5 mm sieve, and the portion that dripped through the sieve was collected by a pan placed underneath the sieve. The segregation index (SI) was determined as the percentage of the portion that dripped through the sieve, as follows:

SI = (*M_P_*/*M_C_*) × 100% (1)

where *M*_P_ is the mass of concrete dripping through the sieve and *M*_C_ is the mass of concrete poured onto the sieve. A low SI indicates high segregation stability (more cohesive), while a high SI indicates low segregation stability (less cohesive).

### 3.3. Compressive Strength Measurement

The specimen preparation and compressive strength test were conducted in accordance with EN 12390-2: 2019 [57] and EN 12390-3: 2019 [58]. For each concrete mix, three 150 mm cubes were produced for compressive strength measurement at ages of 28 days and 70 days, and the mean strength of the cubes that were tested at the same age was taken as the result.

### 3.4. Sorptivity Coefficient Measurement

The sorptivity test was conducted according to ASTM C1585-13 [59] in order to determine the sorptivity coefficient as an indication of the durability. The cubes for testing were oven-dried, and then had side faces coated with epoxy and the bottom face in contact with water. The absorbed water per unit area of bottom face *I* was recorded from time to time for at least five days. Typically, *I* increased linearly with the square root of time *t*, as per the equation below, where *k* is the sorptivity coefficient (mm/s^0.5^):

*I* = *k*(*t*)^0.5^ (2)

The value of *k* was determined as the slope of the best-fit line of *I* versus (*t*)^0.5^ curve. For each concrete mix, three cubes were produced for sorptivity coefficient measurement and the mean value was taken as the result.

## 4. Testing Results

### 4.1. Fresh Properties 

**Figure 2:**
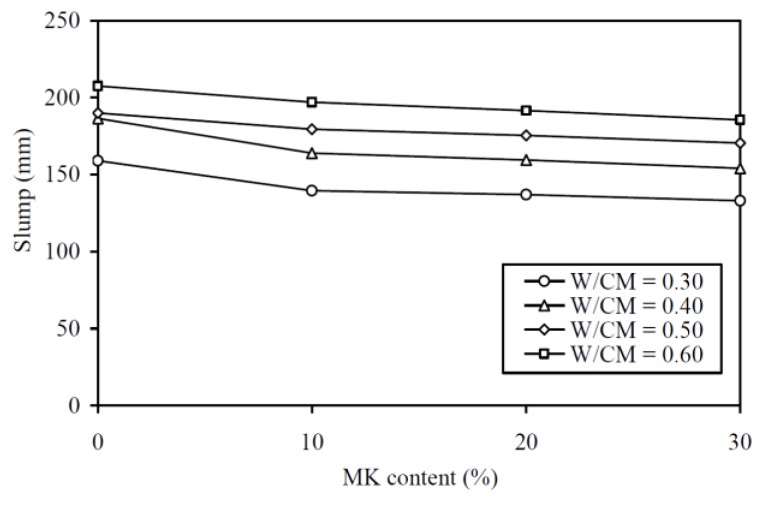
Slump versus metakaolin (MK) content at various W/CM ratios.

The slump results are plotted against the MK contents at various W/CM ratios in Figure 2 and tabulated in the second column of Table A1 in the Appendix for easy capturing of exact values of data. It is seen that the curve for a higher W/CM ratio is generally higher in the figure, showing that, regardless of the MK content, a MK concrete with higher W/CM ratio always has higher deformability. More importantly, it is also seen that within the range of W/CM ratios covered in this study, the addition of MK decreased the deformability. Nonetheless, the decrease in slump with increase in MK content was quite gentle. Particularly, the addition of 30% MK only lowered the slump by 16.4%, 17.4%, 10.3%, and 10.6% at W/CM ratios of 0.30, 0.40, 0.50, and 0.60, respectively. These results are in close agreement with Muduli and Mukharjee [14] who reported that the addition of MK slightly reduced the workability of recycled coarse aggregate concrete. On average, the addition of 30% MK rendered an approximately 14% decrease in the slump.

**Figure 3:**
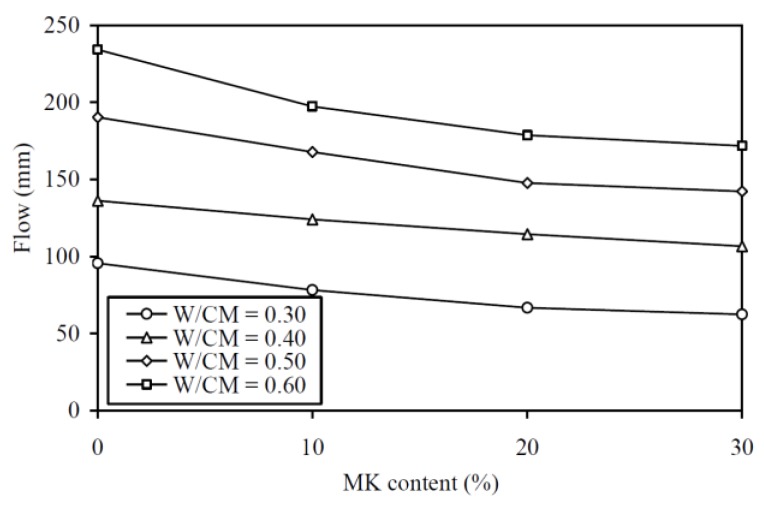
Flow versus MK content at various W/CM ratios.

The flow results are plotted against the MK contents at various W/CM ratios in Figure 3 and tabulated in the third column of Table A1. Again, the curve for a higher W/CM ratio is generally higher in the figure, showing that a MK concrete with higher W/CM ratio always has higher flowability. More importantly, within the range of W/CM ratio covered in this study, the addition of MK decreased the flowability, but the actual decrease in flow with increase in MK content was only moderate, albeit the percentage decrease in flow was larger than the respective percentage decrease in slump. For instance, the addition of 30% MK lowered the flow by 34.7%, 21.6%, 25.2%, and 26.6% at W/CM ratios of 0.30, 0.40, 0.50, and 0.60, respectively. On average, the addition of 30% MK rendered an approximately 27% decrease in the flow.

**Figure 4:**
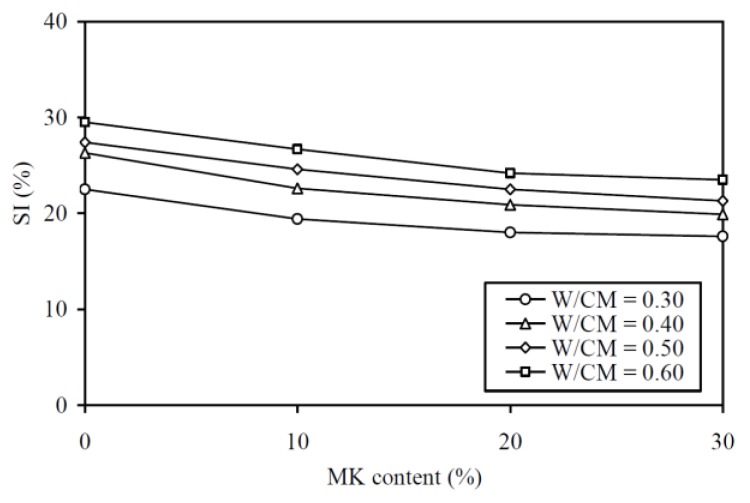
SI versus MK content at various W/CM ratios.

The SI results are plotted against the MK contents at various W/CM ratios in Figure 4 and are tabulated in the fourth column of Table A1 in the Appendix for easy capturing of exact values of data. It is noted that the curve for a higher W/CM ratio is generally higher in the figure, showing that a MK concrete with higher W/CM ratio always has higher SI, or, in other words, lower segregation stability. More importantly, it is evident that within the range of W/CM ratios covered in this study, the addition of MK lowered the SI. Basically, the addition of 30% MK lowered the SI by 21.8%, 24.3%, 22.3%, and 20.3% at W/CM ratios of 0.30, 0.40, 0.50, and 0.60, respectively. On average, the addition of 30% MK lowered the SI by about 22%.

Overall, no significant segregation was noticed during the slump-flow tests. In fact, the test results revealed that the addition of MK improved cohesiveness at the expense of lower deformability and flowability.

### 4.2. Strength

**Figure 5:**
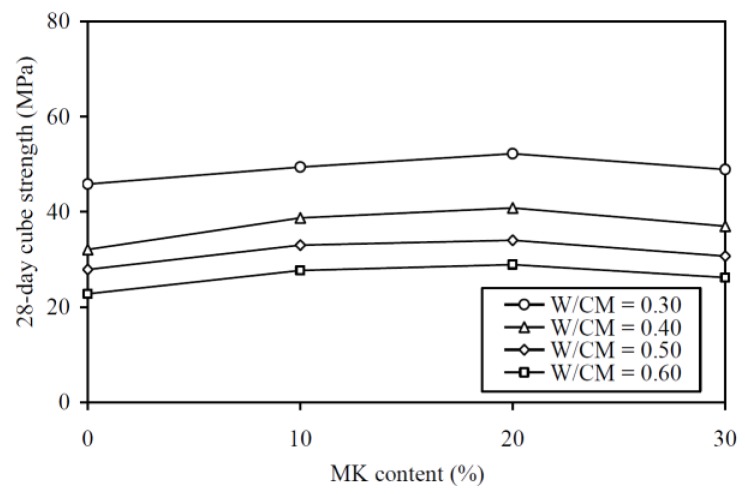
28-day cube strength versus MK content at various W/CM ratios.

The 28-day cube strength results are plotted against the MK contents at various W/CM ratios in Figure 5 and are tabulated in the fifth column of Table A1 in the Appendix for easy capturing of exact values of data. As expected, the curve for a higher W/CM ratio is generally lower in the figure, showing that a MK concrete with higher W/CM ratio always has lower 28-day cube strength. More importantly, at the same W/CM ratio, the addition of MK up to 20% significantly increased the 28-day cube strength, while further addition of MK to 30% turned to slightly decrease the 28-day cube strength. These results are somehow contradictory with Hassan et al. [12] and Valipour et al. [19,20], who reported that the addition of MK always increased the compressive strength. Basically, the addition of 20% MK increased the 28-day cube strength by 14.0%, 27.1%, 21.9%, and 26.8% at W/CM ratios of 0.30, 0.40, 0.50, and 0.60, respectively. On average, the addition of 20% MK increased the 28-day cube strength by approximately 22%. These results are in close agreement with Shen et al. [10], who reported that incorporating 10% to 25% MK as cement replacement resulted in comparable percentage increases in 28-day compressive strength.

**Figure 6:**
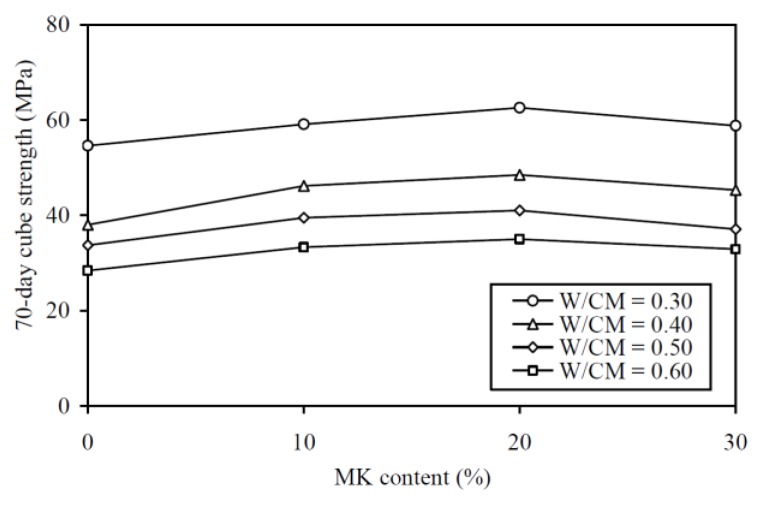
70-day cube strength versus MK content at various W/CM ratios.

The 70-day cube strength results are plotted against the MK contents at various W/CM ratios in Figure 6 and are tabulated in the sixth column of Table A1. Similar to the trend of 28-day cube strength, the curve for a higher W/CM ratio is generally lower in the figure, showing that MK concrete with higher W/CM ratio always has lower 70-day cube strength. More importantly, at the same W/CM ratio, the addition of MK up to 20% significantly increased the 70-day cube strength, while further addition of MK to 30% turned to slightly decrease the 70-day cube strength. This is similar to the variation of the 28-day cube strength with the MK content. Specifically, the addition of 20% MK increased the 70-day cube strength by 14.7%, 27.6%, 21.7%, and 23.2% at W/CM ratios of 0.30, 0.40, 0.50, and 0.60, respectively. On average, the addition of 20% MK increased the 70-day cube strength by approximately 22%.

The 28-day and 70-day cube strength results revealed that the beneficial effect of MK on cube strength gradually diminished when more than 20% MK was added. For the W/CM ratios that were covered in this study, 20% was the optimal MK content for maximum increase in strength. This could be explained by two major phenomena: (1) the MK reacted with the calcium hydroxide produced from cement hydration to form secondary C-S-H gel, but such pozzolanic effect has a certain limit, depending on the MK/cement content ratio; and, (2) the MK particles filled into the interstitial space between the cement particles and densified the micro-structure, but this micro-filling effect was only effective when the MK particles could still be accommodated in the interstitial space. When the MK content was higher than the optimal content, the pozzolanic and micro-filling effects offered diminishing return in strength, despite the further addition of MK.

Regarding the effect of MK at different W/CM ratios, it is noted that the percentage increases in both the 28-day and 70-day cube strengths due to the addition of MK were generally larger at a higher W/CM ratio. This might be explained by the general phenomenon that the tendency to segregate and bleed is higher at higher W/CM ratio, so that the effect of MK in suppressing segregation and bleeding is more apparent at higher W/CM ratio. As segregation and bleeding could cause non-uniform distribution of the free water and thus significant decrease in strength, the addition of MK should have a more significant beneficial effect on strength at a higher W/CM ratio.

### 4.3. Sorptivity

**Figure 7:**
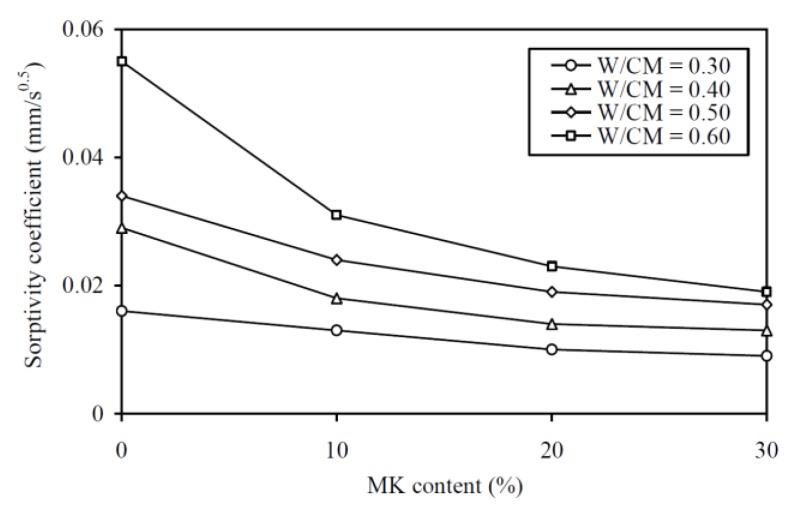
Sorptivity coefficient versus MK content at various W/CM ratios.

The sorptivity coefficient results are plotted against the MK contents at various W/CM ratios in Figure 7 and are tabulated in the last column of Table A1 in the Appendix for easy capturing of exact values of data. As expected, the curve for a higher W/CM ratio is generally higher in the figure, showing that a MK concrete with higher W/CM ratio always has a higher sorptivity coefficient. More importantly, at the same W/CM ratio, the addition of MK up to 30% substantially decreased the sorptivity coefficient, and the decrease in sorptivity tended to be larger at a higher W/CM ratio. Specifically, the addition of 30% MK decreased the sorptivity coefficient by 43.8%, 55.2%, 50.0%, and 65.5% at W/CM ratio of 0.30, 0.40, 0.50 and 0.60, respectively. On average, the addition of 30% MK rendered approximately 54% decrease in sorptivity coefficient. Apparently, the effect of MK addition on sorptivity was much more substantial than those on workability and strength by proportion. This was quite possibly due to the micro-filling effect of the MK particles, which filled into the voids between the cement particles to reduce the voids volume right at the start before the formation of gel products by the hydration and pozzolanic reactions to fill the voids.

## 5. Evaluation of CEFs

### 5.1. Extension of CEF to Fresh Properties

The notion of CEF has been incorporated in industrial standards, such as EN 206 [60] and PD CEN/TR 16639 [61], and investigated by various researchers. Papadakis and Tsimas [30] reported that the CEF of low-calcium fly ash was 2.5 for chloride resistance and 0.5 for carbonation resistance, while the CEF of high-calcium fly ash was 2.0 for chloride resistance and 0.7 for carbonation resistance. Wong and Razak [31] reported that the CEF of MK varied between 1.6 and 2.3 for 28-day strength, and between 1.8 and 4.0 for 180-day strength; whereas, the CEF of micro-silica varied between 2.1 and 3.1 for 28-day strength, and between 2.4 and 3.3 for 180-day strength. Aponte et al. [32] reported that the CEF of fly ash varied between 0.2 and 2.6 for chloride diffusion. Latha et al. [33] reported that the CEFs for strength of slag and fly ash increased with age. Gruyaert et al. [34] compared the CEF concept and the equivalent performance concept of the durability of blastfurnace slag concrete following the Belgian National Standard NBN B 15–100 [62]. Antiohos et al. [35] showed that the CEF of rice husk ash was 0.8 for 28-day strength. Lollini et al. [36] reported that the CEFs of fly ash and slag were approximately 1.5 for durability. Chu and Kwan [11] demonstrated that the presence of MK increased the CEF of micro-silica for strength through synergistic effects. Li et al. [37,38] demonstrated that the CEF of micro-silica was between 2.07 and 2.80 for strength, between 2.78 to 9.87 for chloride resistance and between 3.34 and 6.50 for sulphate resistance; and, the CEF of nano-silica was between 4.30 and 6.05 for strength, between 4.76 to 12.01 for chloride resistance and between 6.75 and 22.06 for sulphate resistance.

However, up to now, the concept of CEF has been applied only to the strength and durability, and not to the fresh properties. Herein, this concept is extended to also apply to the workability in terms of slump and flow and to the cohesiveness in terms of SI. Since the workability and cohesiveness are un-related to the cementing efficiency of the SCM, the term “cementing efficiency factor” is changed to “cement equivalent factor”. As for the concept of cementing efficiency factor, it is defined as the equivalent mass of cement per unit mass of SCM added without affecting the performance attribute under investigation and it is also abbreviated as CEF.

The formula proposed by Smith [63] and Hobbs [64], as given by Equation (3) below, is adopted in order to determine the CEFs with respect to the workability, cohesiveness, strength, and durability. Firstly, the correlation between each performance attribute and the W/C ratio was established by regression analysis of the test results of a number of concrete samples with no MK added. Subsequently, for each performance attribute of the concrete samples with different amounts of MK added and from the correlation between the performance attribute and the W/C ratio established above, the equivalent W/C ratio of each concrete sample was calculated. Finally, the CEF of the MK for each performance attribute was worked out through the following equation:

(W/C)_e_ = W/(C + *k*_perf_ × MK) (3)

where (W/C)_e_ is the equivalent W/C ratio, W, C, and MK are the amount of water, cement, and MK per unit volume of concrete, respectively, and *k*_perf_ is the CEF for the performance attribute being analyzed.

### 5.2. Workability and Cohesiveness

**Figure 8:**
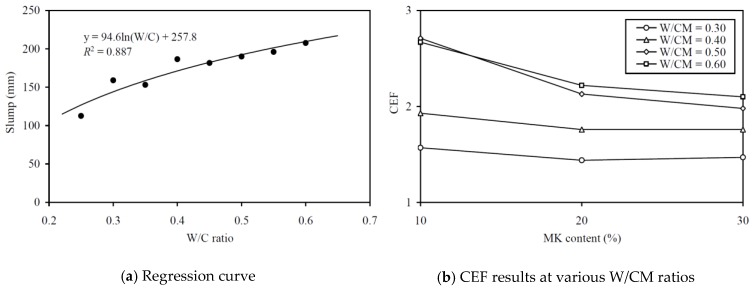
Regression curve and CEF results for slump at various W/CM ratios.

To evaluate the CEF of MK for deformability (in terms of slump), regression analysis for correlating the slump to the equivalent W/C ratio was carried out, as shown in Figure 8a. The regression analysis yielded an *R*^2^ value of 0.887 and the best-fit curve plotted alongside the data points in the figure. This fairly high *R*^2^ value and the closeness of the data points to the best-fit curve proved that the relationship between the slump and effective W/C ratio was applicable for evaluating the slump CEF. The slump CEFs obtained are plotted against the MK contents at various W/CM ratios in Figure 8b and tabulated in the third column of Table A2 in the Appendix for easy capturing of exact values of data. It is seen that the slump CEF of MK varied from 1.44 to 2.71 and it had an average value of 1.98. Somehow, the curve for a higher W/CM ratio is generally higher in the figure, which indicated that the slump CEF of MK is larger at a higher W/CM ratio. It should be noted that a slump CEF of MK larger than 1.0 means that the addition of MK as OPC replacement would decrease the slump, and the larger the value of slump CEF, the greater is the adverse effect on slump.

**Figure 9:**
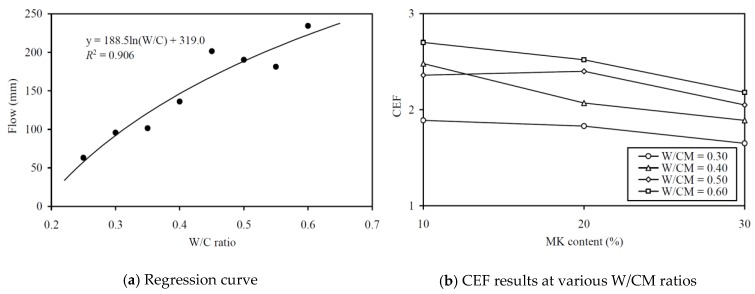
Regression curve and CEF results for flow at various W/CM ratios.

To evaluate the CEF of MK for flowability (in terms of flow), regression analysis for correlating the flow to the equivalent W/C ratio was carried out, as shown in Figure 9a. The regression analysis yielded an *R*^2^ value of 0.906 and the best-fit curve is plotted alongside the data points in the figure. The good correlation proved that the relationship between the flow and effective W/C ratio was applicable for evaluating the flow CEF. The flow CEFs obtained are plotted against the MK contents at various W/CM ratios in Figure 9b and tabulated in the fourth column of Table A2. In general, the flow CEF of MK varied from 1.65 to 2.70 and it had an average value of 2.17. As before, the curve for higher W/CM ratio is generally higher in the figure, which indicated that the flow CEF of MK is larger at a higher W/CM ratio. Again, it should be noted that a flow CEF of MK that is larger than 1.0 means the addition of MK as OPC replacement would decrease the flow, and the larger the value of flow CEF, the greater is the adverse effect on flow.

**Figure 10:**
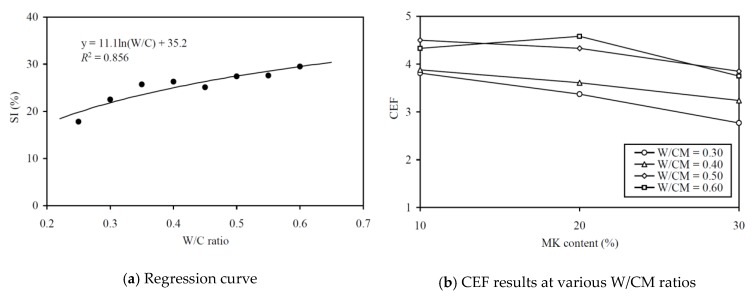
Regression curve and CEF results for SI at various W/CM ratios.

To evaluate the CEF of MK for cohesiveness (in terms of SI), regression analysis for correlating the SI to the equivalent W/C ratio was carried out, as shown in Figure 10a. The regression analysis yielded an *R*^2^ value of 0.856 and the best-fit curve is plotted alongside the data points in the figure. The good correlation proved that the relationship between the SI and effective W/C ratio was applicable for evaluating the cohesiveness CEF. The cohesiveness CEFs so obtained are plotted against the MK contents at various W/CM ratios in Figure 10b and tabulated in the fifth column of Table A2 in the Appendix for easy capturing of exact values of data. On the whole, the cohesiveness CEF of MK varied from 2.77 to 4.58 and it had an average value of 3.83. Moreover, as before, the curve for higher W/CM ratio is higher in the figure, which indicated that the cohesiveness CEF of MK tends to be larger at a higher W/CM ratio. The fairly large cohesiveness CEF of well above 2.0 means that the addition of MK as OPC replacement is highly effective in improving the cohesiveness and segregation stability of the concrete produced.

However, it should be noted that the CEFs of MK for the workability attributes and cohesiveness are very likely dependent on the superplasticiser dosage added to the concrete mix. Further research for incorporating the possible effects of superplasticiser dosage on the slump, flow, and cohesiveness CEFs is recommended. It is envisaged that by adding more superplasticiser, the adverse effects of MK on workability could be mitigated and the CEFs for workability could be lowered.

### 5.3. Strength

**Figure 11:**
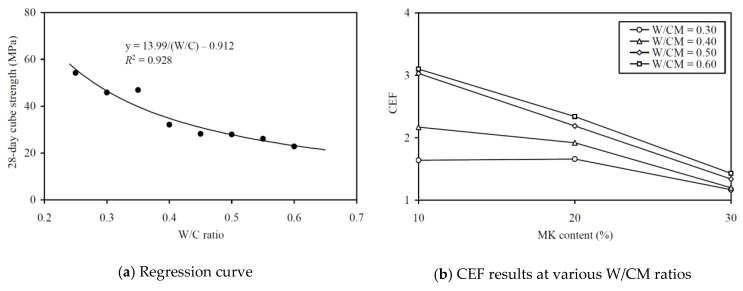
Regression curve and CEF results for 28-day cube strength at various W/CM ratios.

**Figure 12:**
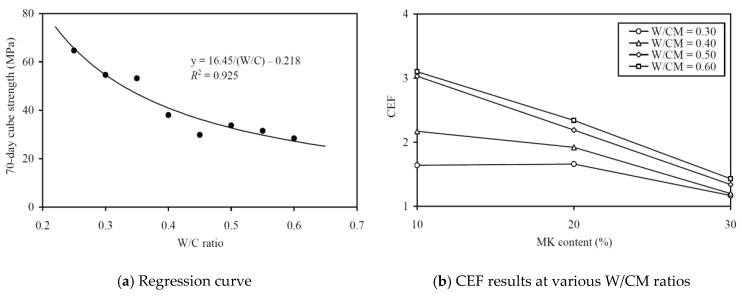
Regression curve and CEF results for 70-day cube strength at various W/CM ratios.

To evaluate the CEFs of MK for strength (in terms of 28-day strength and 70-day strength), regression analysis has been carried out to correlate the 28-day and 70-day strengths to the equivalent W/C ratio, as in Figure 11a and Figure 12a, respectively. The regression analysis yielded *R*^2^ values of 0.928 and 0.925, respectively, proving that the relationships between the 28-day and 70-day strengths and the effective W/C ratio were applicable for evaluating the strength CEFs. The strength CEFs so obtained are plotted against the MK contents at various W/CM ratios in Figure 11b and Figure 12b and tabulated in the sixth and seventh columns of Table A2 in the Appendix. It is seen that the 28-day strength CEF of MK varied from 1.17 to 3.10 and it had an average value of 1.93, whereas the 70-day strength CEF of MK varied from 1.26 to 3.42 and had an average value of 2.12. These results are in close agreement with Wong and Razak [31], who reported that the CEF of MK varied between 1.6 and 2.3 for 28-day strength. Moreover, the strength CEF of MK is generally larger at higher W/CM ratio and tends to be smaller at higher MK content, regardless of whether the strength is measured at 28-day or at 70-day. Hence, the addition of MK is more effective in increasing the 28-day and 70-day strengths at higher W/CM ratio, but the effectiveness gradually decreases as the MK content keeps on increasing.

When comparing the CEFs of MK for 28-day strength and 70-day strength, it is noted that the CEF for 70-day strength is slightly larger than that for 28-day strength. This might be attributed to the relatively slow, but longer lasting, pozzolanic reaction of the MK compared to the hydration reaction of OPC.

### 5.4. Sorptivity

**Figure 13:**
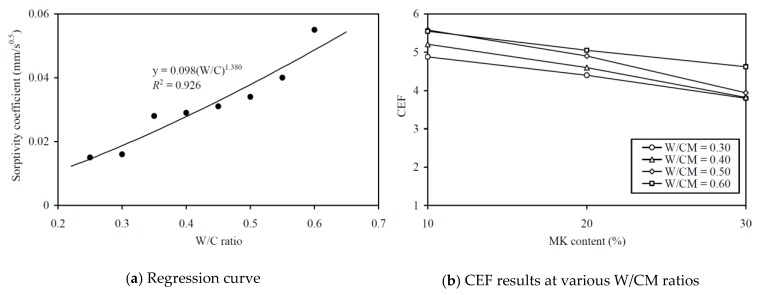
Regression curve and CEF results for sorptivity coefficient at various W/CM ratios.

To evaluate the CEF of MK for sorptivity (in terms of sorptivity coefficient), regression analysis has been carried out to correlate the sorptivity coefficient to the equivalent W/C ratio, as in Figure 13a. A very high *R*^2^ value of 0.926 was obtained, proving that the relationship between the sorptivity coefficient and effective W/C ratio was applicable for evaluating the sorptivity CEF. The sorptivity CEFs so obtained are plotted against the MK contents at various W/CM ratios in Figure 13b and are tabulated in the last column of Table A2 in the Appendix for easy capturing of the exact values of data. These results show that the sorptivity CEF of MK varied from 3.80 to 5.58 and had an average value of 4.70. As for the other performance attributes, the curve for higher W/CM ratio is generally higher in the figure, showing that the sorptivity CEF of MK is generally larger at a higher W/CM ratio. Moreover, at all W/CM ratios, the sorptivity CEF of MK is generally smaller at higher MK content. This is probably due to the gradual saturation of the micro-filling effect of the MK and the diminishing return of the micro-structural densification when the MK added is about to fill up the voids between the larger size particles.

## 6. Concurrently Achieved Performances

Although the addition of MK would improve the cohesiveness, strength, and durability, it would, at the same time, impair the workability (especially in terms of flow). Hence, it is not easy to achieve good cohesiveness, strength and durability performance and high workability at the same time. The cohesiveness, strength, and durability performance and flowability that can be concurrently achieved are examined below.

### 6.1. Concurrently Achieved Cohesiveness and Flowability

**Figure 14:**
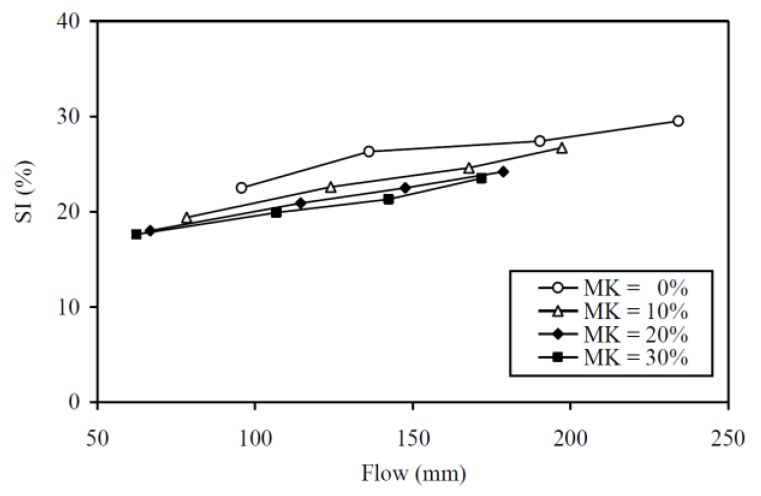
Concurrent cohesiveness and flowability performances at various MK contents.

To illustrate the concurrently achieved cohesiveness and flowability at different MK contents, the cohesiveness is plotted against the flow in Figure 14. The figure shows that an increase in MK content shifts the SI-flow curve downwards, indicating that the addition of MK would, at the same flowability, improve the cohesiveness. This is echoed by the finding that the CEF values for SI are always larger than those for flow. As both cohesiveness and flowability are needed for self-consolidating concrete [65,66,67], such increase in cohesiveness at the same flowability, i.e. improvement in concurrent cohesiveness-flowability performance, due to MK addition, demonstrates that MK is a useful SCM for producing self-consolidating concrete.

### 6.2. Concurrently Achieved Strength and Flowability

**Figure 15:**
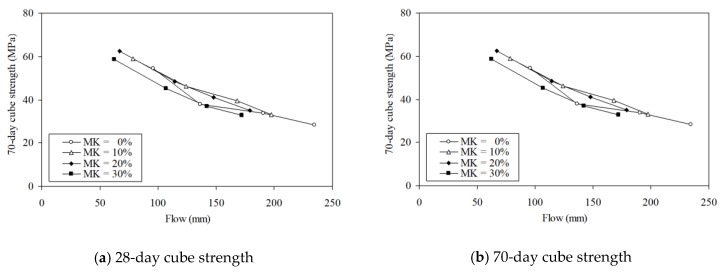
Concurrent strength and flowability performances at various MK contents.

To illustrate the concurrently achieved strength and flowability at different MK contents, the 28-day and 70-day cube strengths are plotted against the flow in Figure 15. The figure shows that the addition of up to 20% MK shifts both the 28-day strength-flow curve and the 70-day strength-flow curve to the left, but neither upwards or downwards. Hence, within the range of MK concrete mixes produced for testing, the addition of up to 20% has little effect on the concurrent strength-flowability performance. This is echoed by the finding that the CEF values for flow and those for strength are quite close to each other at MK content not higher than 20%. However, the addition of 30% MK shifts both the 28-day strength-flow curve and the 70-day strength-flow curve to the left and slightly downwards. Hence, within the range of MK concrete mixes produced for testing, the addition of 30% MK has a slight adverse effect on the concurrent strength-flowability performance. This is echoed by the finding that, at MK content equal to 30%, the CEF values for flow are higher than those for strength. This might be a bit disappointing, but such an adverse effect on concurrent strength-flowability performance could be avoided by limiting the MK content to at most 20%.

While interpreting the above results, it should be noted that the effect of MK on the workability is dependent on the superplasticiser dosage. Theoretically, the addition of more superplasticiser could improve the concurrent strength-workability performance. If, by adding more superplasticiser, the workability CEFs of the MK could be lowered to approximately the same as the strength CEFs, then the concurrent strength-workability performance would not be downgraded. Additionally, if the workability CEFs of the MK could be lowered to lower than the strength CEFs, then the concurrent strength-workability performance could even be upgraded. Further research on the effect of superplasticiser dosage on the workability CEFs and the concurrent strength-workability performance is recommended. The concept of workability CEFs developed in this research should be a useful tool for this kind of work.

### 6.3. Concurrently Achieved Durability and Flowability

**Figure 16:**
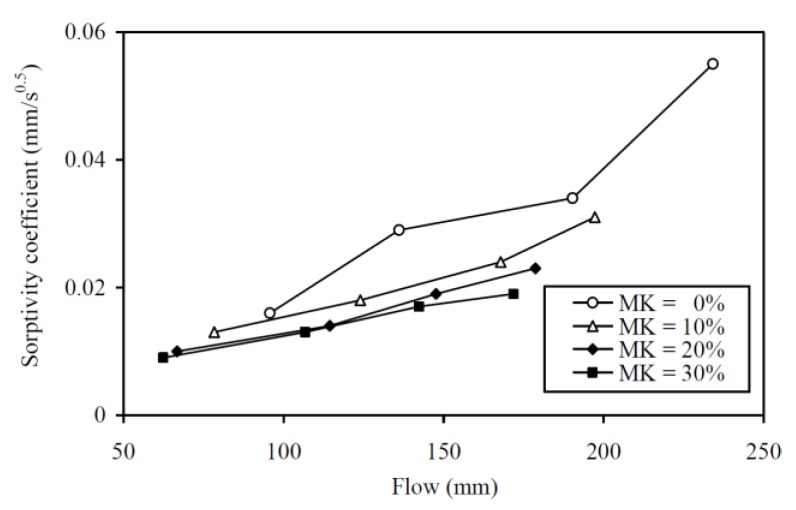
Concurrent sorptivity coefficient and flowability performances at various MK contents.

To illustrate the concurrently achieved durability and flowability at different MK contents, the sorptivity coefficient is plotted against the flow in Figure 16. The figure shows that an increase in MK content always shifts the sorptivity coefficient-flow curve downwards, which indicates that the addition of MK would, at the same flowability, decrease the sorptivity to improve the durability. Hence, within the range of MK concrete mixes produced for testing, the addition of up to 30% MK would improve the concurrent durability-flowability performance. This is echoed by the finding that the CEF values for sorptivity are always larger than those for flow. However, it should be noted that sorptivity is only one of the durability performance attributes. For different durability performance attributes, such as sulphate, carbonation, and chloride resistances, the CEFs could be very different [44]. Further research on the CEFs of MK for the other durability performance attributes is recommended.

### 6.4. Overall Remarks on the CEFs of MK

The above findings demonstrated that all of the CEFs of MK for workability, cohesiveness, strength, and durability are larger than unity. The high cement equivalence of the MK is due to the high fineness and pozzolanic reactivity of the MK. However, such high cement equivalence of the MK is a two-edge sword. The high workability CEFs would cause certain adverse effects on the workability and, when the workability CEFs are larger than the strength CEFs, the concurrent strength-workability performance would be impaired. This may be alleviated by increasing the superplasticiser dosage. As the fineness of solid ingredients has certain effects on the workability, it is anticipated that the fineness of the MK would also have some effects on the workability CEFs. On the other hand, the high strength and sorptivity CEFs indicate that the MK is a highly effective SCM for improving the strength and durability.

Last but not least, this study has demonstrated that the concept of CEF might be extended to fresh properties and the CEFs for the various performance attributes are useful parameters for quantifying the effectiveness of a SCM. In concrete mix design and optimization [61], the CEFs of the available SCMs for different performance attributes are very useful data for the direct determination of the suitable ranges of the mix parameters, so that the number of trial mixing for concrete mix design could be minimized. Further research to develop a concrete mix design method based on the CEFs is highly recommended.

## 7. Conclusions

The concept of cementing efficiency has been extended to cement equivalence for more general application to all performance attributes, including the fresh properties, such as workability and cohesiveness. To explore such possible application of cement equivalent factors (CEFs) to metakaolin (MK), a systematic research programme encompassing a total of 20 concrete mixes with various MK contents added as replacement of ordinary Portland cement (OPC) and water/cementitious materials (W/CM) ratios has been completed. The research findings are summarized, as follows:

(1) The addition of up to 20% MK as OPC replacement best improved the 28-day and 70-day cube strengths; whereas, the addition of up to 30% MK as OPC replacement always improved the cohesiveness (in terms of segregation index) and durability (in terms of sorptivity), but impaired the workability (in terms of slump and flow).
(2) Regarding the fresh properties, the ranges of CEFs of MK were 1.44 to 2.71, 1.65 to 2.70, and 2.77 to 4.58 for slump, flow, and cohesiveness, respectively. The corresponding average CEFs were 1.98, 2.17, and 3.83, respectively. It is noteworthy that the CEF for cohesiveness was larger than those for workability.
(3) Regarding the strength, the ranges of CEFs of MK were 1.17 to 1.93 and 1.26 to 2.12 for 28-day and 70-day cube strengths, respectively. The corresponding average CEFs were 1.93 and 2.12, respectively. As expected, the CEF for 70-day strength was slightly higher than that for 28-day strength.
(4) Regarding the durability, the ranges of CEFs of MK was 3.80 to 5.58 for the sorptivity coefficient, and the corresponding average CEF was as high as 4.70, which indicated that the MK is highly effective in reducing the sorptivity.
(5) For all the performance attributes including workability, cohesiveness, strength and durability, all the CEFs of MK were larger than unity and generally increased with increasing W/CM ratio and decreased with increasing MK content.
(6) The concurrently achieved performances showed that the MK was a highly effective cementitious material for improving the cohesiveness, strength, and durability, albeit it could reduce the workability.

The experience gained in this study demonstrated that the concept of CEF can be extended to encompass the fresh properties and the CEFs are useful parameters for quantifying the influence of the SCM on the various performance attributes. The CEFs so obtained would help in determining the plausible ranges of MK content in concrete and relieve the time and effort needed for performing trial mixing in practical concrete mix design. The above are the major scientific contributions of this paper. Further research on harmonizing the ranges of CEFs of various SCMs for different performance attributes is advocated. Particularly, since the CEFs for fresh properties are affected by the superplasticiser dosage, the effects of the superplasticiser dosage should be included in the further research.

## Figures and Tables

**Table 1:** Chemical compositions of the cementitious materials.

Chemical Composition	OPC (%)	MK (%)
SiO_2_	23.9	51.7
CaO	62.3	0.5
Al_2_O_3_	4.9	44.1
Fe_2_O_3_	3.7	0.9
MgO	2.4	< 0.3
TiO_2_	-	1.4
Na_2_O	< 0.3	< 0.2
K_2_O	0.4	< 0.1
Loss on ignition	1.9	0.6

**Table 2:** Mix proportions of concrete.

Mix No. (M-V_MK_- W/CM)	OPC (kg/m^3^)	MK (kg/m^3^)	Fine Aggregate (kg/m^3^)	Coarse Aggregate (kg/m^3^)	Water (kg/m^3^)	Super- Plasticiser (kg/m^3^)
M-0-0.25	663.7	0.0	634.0	951.0	146.0	19.9
M-0-0.30	610.4	0.0	634.0	951.0	164.8	18.3
M-10-0.30	553.7	51.4	634.0	951.0	163.4	18.2
M-20-0.30	496.2	103.6	634.0	951.0	161.9	18.0
M-30-0.30	437.7	156.7	634.0	951.0	160.5	17.8
M-0-0.35	565.0	0.0	634.0	951.0	180.8	16.9
M-0-0.40	525.9	0.0	634.0	951.0	194.6	15.8
M-10-0.40	477.7	44.3	634.0	951.0	193.1	15.7
M-20-0.40	428.5	89.5	634.0	951.0	191.7	15.5
M-30-0.40	378.5	135.5	634.0	951.0	190.2	15.4
M-0-0.45	491.9	0.0	634.0	951.0	206.6	14.8
M-0-0.50	462.0	0.0	634.0	951.0	217.1	13.9
M-10-0.50	420.0	39.0	634.0	951.0	215.7	13.8
M-20-0.50	377.1	78.8	634.0	951.0	214.3	13.7
M-30-0.50	333.4	119.4	634.0	951.0	212.8	13.6
M-0-0.55	435.5	0.0	634.0	951.0	226.5	13.1
M-0-0.60	411.9	0.0	634.0	951.0	234.8	12.4
M-10-0.60	374.7	34.8	634.0	951.0	233.4	12.3
M-20-0.60	336.7	70.3	634.0	951.0	232.0	12.2
M-30-0.60	297.9	106.7	634.0	951.0	230.6	12.1

## Appendix A

**Table A1:** Testing results.

Mix no. (M-V_MK_- W/CM)	Slump (mm)	Flow (mm)	SI (%)	28-Day Strength (MPa)	70-Day Strength (MPa)	Sorptivity Coefficient (mm/s^0.5^)
M-0-0.25	112.5	63.1	17.8	54.2	64.7	0.015
M-0-0.30	159.0	95.7	22.5	45.8	54.6	0.016
M-10-0.30	139.5	78.3	19.4	49.4	59.1	0.013
M-20-0.30	137.0	66.7	18.0	52.2	62.6	0.010
M-30-0.30	133.0	62.4	17.6	48.9	58.8	0.009
M-0-0.35	153.0	101.4	25.7	46.9	53.2	0.028
M-0-0.40	186.5	136.1	26.3	32.1	38.0	0.029
M-10-0.40	164.0	124.0	22.6	38.7	46.2	0.018
M-20-0.40	159.5	114.4	20.9	40.8	48.5	0.014
M-30-0.40	154.0	106.7	19.9	37.0	45.3	0.013
M-0-0.45	181.5	201.3	25.1	28.2	29.8	0.031
M-0-0.50	190.0	190.3	27.4	27.9	33.7	0.034
M-10-0.50	179.5	167.8	24.6	33.0	39.5	0.024
M-20-0.50	175.5	147.6	22.5	34.0	41.0	0.019
M-30-0.50	170.5	142.3	21.3	30.7	37.1	0.017
M-0-0.55	196.0	181.2	27.6	26.1	31.5	0.040
M-0-0.60	207.5	234.2	29.5	22.8	28.4	0.055
M-10-0.60	197.0	197.3	26.7	27.7	33.3	0.031
M-20-0.60	191.5	178.7	24.2	28.9	35.0	0.023
M-30-0.60	185.5	171.8	23.5	26.2	32.9	0.019

**Table A2:** Cement equivalent factors.

MK Content (%)	W/CM Ratio	CEF					
		Slump	Flow	SI	28-Day Strength	70-Day Strength	Sorptivity
10	0.30	1.57	1.89	3.81	1.64	1.87	4.88
	0.40	1.93	2.48	3.88	2.17	2.40	5.21
	0.50	2.71	2.36	4.50	3.03	3.27	5.58
	0.60	2.67	2.70	4.33	3.10	3.42	5.55
20	0.30	1.44	1.83	3.37	1.66	1.80	4.40
	0.40	1.76	2.07	3.61	1.92	2.01	4.60
	0.50	2.13	2.40	4.33	2.19	2.39	4.90
	0.60	2.22	2.52	4.58	2.34	2.54	5.05
30	0.30	1.47	1.65	2.77	1.17	1.26	3.80
	0.40	1.76	1.89	3.24	1.20	1.36	3.82
	0.50	1.98	2.05	3.83	1.34	1.45	3.94
	0.60	2.10	2.18	3.75	1.43	1.72	4.62
Minimum		1.44	1.65	2.77	1.17	1.26	3.80
Maximum		2.71	2.70	4.58	3.10	3.42	5.58
Average		1.98	2.17	3.83	1.93	2.12	4.70
